# Supplementary material for: The Systems Biology Research Tool: evolvable open-source software
Source: BMC Syst Biol. 2008 Jun 29;2:55. doi: 10.1186/1752-0509-2-55 (PMC2446383; doi:10.1186/1752-0509-2-55)
Supplement: Additional file 1 — SBRT Archive. An archive of the current version of the Systems Biology Research Tool. [file 1752-0509-2-55-S1.zip › sbrt-1.4.0/doc/developers_guide/api/sbrt/kernel/fba/proc/NetworkInfoProcess.Results.html]

NetworkInfoProcess.Results


|  |  |  |
| --- | --- | --- |
| **Overview** \| **Package** \| **Class** \| **Use** \| **Tree** \| **Deprecated** \| **Index** \| **Help** | |  |
| **PREV CLASS**   **NEXT CLASS** | **FRAMES**    **NO FRAMES**     **All Classes** |  |
| SUMMARY: NESTED \| FIELD \| CONSTR \| METHOD | DETAIL: FIELD \| CONSTR \| METHOD |  |


---


## sbrt.kernel.fba.proc Class NetworkInfoProcess.Results

```
java.lang.Object
  sbrt.kernel.fba.proc.NetworkInfoProcess.Results
```

**Enclosing class:**: NetworkInfoProcess

---

``` public static class NetworkInfoProcess.Results extends java.lang.Object ```

This class is used to represent the information gathered about a
fluxome.

**Author:**
:   This class was written and documented by Jeremiah Wright
    while in the Wagner lab.

---

| **Method Summary** | |
| --- | --- |
| `java.util.Set<java.lang.String>` | `getCatalysts()`             Returns the catalysts contained in the fluxome. |
| `java.util.Set<java.lang.String>` | `getChemSpecies()`             Returns the metabolites contained in the fluxome. |
| `java.util.Map<java.lang.String,java.util.Set<java.lang.String>>` | `getEnzymeRxnAssoc()`             Returns the catalyst-reaction associations contained in the fluxome. |
| `java.util.Collection<java.util.Set<java.lang.String>>` | `getEquivRxns()`             Returns the equivalent reactions contained in the fluxome. |
| `java.util.Set<java.lang.String>` | `getRxnNames()`             Returns the names of all reactions contained in the fluxome. |
| `SparseDoubleMatrix<java.lang.String,java.lang.String>` | `getStoichMatrix()`             Returns the stoichiometry matrix of the fluxome. |

**Methods inherited from class java.lang.Object**

`clone, equals, finalize, getClass, hashCode, notify, notifyAll, toString, wait, wait, wait`

**Method Detail**

### getChemSpecies

```
public java.util.Set<java.lang.String> getChemSpecies()
```

:   Returns the metabolites contained in the fluxome.

    :   **Returns:**: the metabolites contained in the fluxome.

---


### getRxnNames

```
public java.util.Set<java.lang.String> getRxnNames()
```

:   Returns the names of all reactions contained in the fluxome.

    :   **Returns:**: the names of all reactions contained in the fluxome.

---


### getCatalysts

```
public java.util.Set<java.lang.String> getCatalysts()
```

:   Returns the catalysts contained in the fluxome.

    :   **Returns:**: the catalysts contained in the fluxome.

---


### getEquivRxns

```
public java.util.Collection<java.util.Set<java.lang.String>> getEquivRxns()
```

:   Returns the equivalent reactions contained in the fluxome.

    :   **Returns:**: the equivalent reactions contained in the fluxome.

---


### getEnzymeRxnAssoc

```
public java.util.Map<java.lang.String,java.util.Set<java.lang.String>> getEnzymeRxnAssoc()
```

:   Returns the catalyst-reaction associations contained in the fluxome.

    :   **Returns:**: catalysts mapped to the names of the reactions they catalyze.

---


### getStoichMatrix

```
public SparseDoubleMatrix<java.lang.String,java.lang.String> getStoichMatrix()
```

:   Returns the stoichiometry matrix of the fluxome.

    :   **Returns:**: the stoichiometry matrix of the fluxome.


---


|  |  |  |
| --- | --- | --- |
| **Overview** \| **Package** \| **Class** \| **Use** \| **Tree** \| **Deprecated** \| **Index** \| **Help** | |  |
| **PREV CLASS**   **NEXT CLASS** | **FRAMES**    **NO FRAMES**     **All Classes** |  |
| SUMMARY: NESTED \| FIELD \| CONSTR \| METHOD | DETAIL: FIELD \| CONSTR \| METHOD |  |


---
